# From Charcuterie to Plant-Based: Harnessing *Penicillium nalgiovense* for Innovative Soybean Co-Culture Fermentation

**DOI:** 10.3390/foods15061053

**Published:** 2026-03-17

**Authors:** Xin Hui Chin, Ryan Soh, Geraldine Chan, Pnelope Ng, Aaron Thong, Hosam Elhalis, Yoganathan Kanagasundaram, Yvonne Chow, Shao Quan Liu

**Affiliations:** 1 Singapore Institute of Food and Biotechnology Innovation, Agency for Science, Technology and Research, 31 Biopolis Way, Nanos, Singapore 138669, Singapore; chin_xin_hui@a-star.edu.sg (X.H.C.);; 2 Department of Food Science and Technology, Faculty of Science, National University of Singapore, 2 Science Drive 2, Singapore 117543, Singapore; 3 Sydney Technical Centre, AB Mauri, 1 Richardson Place, North Ryde, NSW 2113, Australia

**Keywords:** *Penicillium nalgiovense*, microbial fermentation, mixed cultures, soybean

## Abstract

Improving the flavour of soybean-based ingredients remains challenging as soybeans naturally contain compounds that generate green and beany notes. This study evaluated how the surface-growing food-grade fungus *Penicillium nalgiovense* (PN), alone and together with selected yeasts and lactic acid bacteria, alters the chemistry and sensory attributes of soybeans during solid-state fermentation. PN showed strong proteolytic activity in the monoculture fermentation, producing the highest accumulation of free amino acids (1324 mg/100 g), while its combination with *Lactiplantibacillus plantarum* (LP) further increased this to 1487 mg/100 g due to acid-assisted protease action. Sugar and organic acid profiles reflected distinct metabolic roles among the strains; for example, PNLP and PN-*Debaryomyces hansenii* (DH) depleted sucrose and glucose completely by 72 h, whereas DH retained substantial sucrose. Fermentation also altered the lipid profiles, where PN-*Kluyveromyces marxianus* (KM) showed the highest increase in polyunsaturated fatty acids, with linoleic and α-linolenic acid increasing more than twofold and threefold, respectively. Volatile analysis showed a significant decrease in hexanal (from 18.3 µg/g in control to <2.0 µg/g post fermentation) and an increase in esters, floral alcohols, and savoury compounds depending on the microbial pairing. Electronic tongue profiling showed that PN-fermented samples produced the strongest savoury taste signals. Overall, the work highlights how specific PN-yeast or PN-LAB combinations can be used to modulate flavour development in fermented soy-based substrates.

## 1. Introduction

Growing concerns regarding the environmental footprint of conventional livestock production and the need for nutritionally robust and sustainable protein sources have prompted renewed interest in alternatives to animal-derived foods [1,2]. Plant proteins and microbial processing systems are increasingly viewed as viable contributors to future protein supply chains. Among emerging strategies, the application of filamentous fungi to plant substrates has attracted substantial attention due to its capacity to enhance nutritional functionality while improving sensory attributes [3,4].

Fungal fermentation has long been utilised in traditional food systems, where moulds such as *Aspergillus* and *Rhizopus* transform cereals and legumes through enzymatic activity. Beyond structural modification, filamentous fungi possess diverse proteolytic, lipolytic, and carbohydrase systems that liberate amino acids, bioactive peptides, vitamins, and minerals from complex plant matrices [4]. Increasingly, fungal solid-state fermentation (SSF) is being explored as a targeted strategy to upgrade plant proteins, reduce anti-nutritional factors, and diversify flavour-active metabolites in legumes and other plant-based substrates [3,5]. Such approaches are particularly relevant for improving the sensory quality of plant-based protein ingredients, where flavour limitations often hinder consumer acceptance.

Soybean *(Glycine max)* exemplifies a crop that is highly nutritious yet underutilised as a fermentation substrate. Its high protein content, favourable amino acid profile, dietary fibre, and micronutrient composition make it a strong candidate for plant-based ingredient development [6]. However, raw soybeans contain anti-nutritional components such as phytic acid and trypsin inhibitors that impair nutrient bioavailability [7]. In addition, characteristic “beany” and “grassy” volatiles, largely derived from lipid oxidation pathways, limit sensory appeal [5]. Fermentation offers an effective means of addressing these challenges. Soybeans contain fermentable carbohydrates, including sucrose, raffinose, stachyose, glucose, and fructose, that readily support fungal growth and enzymatic metabolism [8]. During SSF, fungal enzymes hydrolyse storage proteins, carbohydrates, and lipids, reducing anti-nutritional factors, modifying volatile profiles, and improving digestibility.

Traditionally, whole-soybean fermentations have relied on moulds such as *Rhizopus*, *Aspergillus*, and *Fusarium*. Despite being widely adopted in Asian diets for centuries, traditionally fermented soybean products are still not commonly incorporated into the Western diet as a protein alternative. This is partly due to flavour familiarity and cultural preferences, but also to safety concerns associated with certain strains. Some species and isolates of *Aspergillus* and *Fusarium* are known producers of mycotoxins such as aflatoxins, ochratoxin A, and fumonisins [9,10]. Although controlled starter cultures mitigate this risk, the association between mould fermentation and potential production highlights the need for carefully selected and validated alternative fungal cultures that can enhance both safety and sensory quality.

One promising candidate is *Penicillium nalgiovense* (PN), a filamentous mould widely used in dry-cured meat fermentation. PN is characterised by white-to-grey surface mycelium and is valued for its ability to contribute to flavour development, lipid transformation, and microbial stability in sausages and hams [11]. While isolated reports have described toxigenic PN strains [12], commercial starter culture strains have been demonstrated to be non-toxigenic and even protective against undesirable mould growth [13,14]. Despite its established role in meat fermentation, the behaviour of PN in plant-based substrates remains largely unexplored.

It is important to distinguish the present approach from traditional tempeh-type fermentations. Tempeh production relies primarily on *Rhizopus* spp., which rapidly colonise soybeans and form a dense mycelial matrix that binds cotyledons into a cohesive structure. In such systems, structural transformation and improved digestibility are primary objectives, while flavour modulation is secondary. In contrast, the present study investigates PN as a functional starter culture for flavour-driven biochemical modulation of soybean substrates. Unlike *Rhizopus*, PN exhibits distinct proteolytic and lipolytic capabilities and is applied here in both monoculture and co-culture systems to influence metabolic interactions and promote the formation of aroma-active compounds.

In this study, we evaluated the behaviour of PN in soybean SSF, both alone and in combination with selected yeasts and lactic acid bacteria. Rather than considering PN solely as a surface mould, we examined how it shapes microbial succession, alters substrate utilisation patterns, and influences the formation of taste- and aroma-active metabolites. Microbial population dynamics, fermentation metabolite, fatty acid, free amino acid, and volatile compound profiles were monitored across monoculture and co-culture systems. By integrating microbial ecology with metabolite profiling, this work aims to clarify whether PN can serves as a functional culture for flavour-directed soybean fermentation and whether PN-based co-cultures offer advantages over conventional mould systems. Collectively, these findings contribute to the rational design of fungal-driven plant fermentations with improved sensory profiles for plant-based protein applications.

## 2. Materials and Methods

### 2.1. Microbial Strains and Culture Preparation

The microorganisms used in this study included the filamentous fungus *Penicillium nalgiovense* (PN), the yeasts *Kluyveromyces marxianus* (KM) and *Debaryomyces hansenii* (DH), and the lactic acid bacterium *Lactiplantibacillus plantarum* (LP). These strains were selected based on their enzymatic potential for carbohydrate metabolism, lipid transformation, and proteolysis during fermentation. The *Penicillim nalgiovense* strain used in this study was obtained from the American Type Culture Collection (ATCC), which provides authenticated reference strains with documented strain history. Commercial PN starter strains used in food fermentations have been reported to undergo screening for major mycotoxins and are not associated with toxin production under controlled food fermentation conditions. To further substantiate strain-level safety under the present fermentation conditions, targeted LC-MS screening of selected Penicillium-associated secondary metabolites was conducted. The compounds analysed included hydroxy-aspergillic acid, neoaspergillic acid, aspergillic acid, and patulin (putative).

Preparation of microbial inocula followed the procedures previously described by [15] for soybean solid-state fermentation. Briefly, yeast strains were propagated in yeast extract–peptone–dextrose (YPD) medium at 30 °C for 24 h under aerobic conditions, while LP was cultivated in MRS broth at 30 °C for 24 h.

PN was maintained on potato dextrose agar (PDA) plates and incubated at 25 °C until sporulation occurred. Spore suspensions were prepared by washing the agar surface with sterile distilled water containing 0.1% (*v*/*v*) Tween-80, following the protocol described in [15].

### 2.2. Preparation of Soybean Substrates

Preparation of the soybean substrates followed the procedure described in [15] with minor modifications. Soybeans were soaked in water at a soybean-to-water ratio of 1:4 (*w*/*v*) supplemented with 0.05% (*w*/*w*) acetic acid (Sigma-Aldrich, St. Louis, MO, USA) to suppress undesirable microbial growth during hydration. Soaking was carried out at 25 °C for 24 h.

After soaking, the beans were drained and rinsed thoroughly with tap water to remove residual acid. The seed coats were subsequently removed manually, achieving a dehulling efficiency greater than 95%. Removal of the hull improves microbial accessibility to the cotyledon and facilitates enzymatic hydrolysis during fermentation.

The moisture content of the prepared soybeans was measured gravimetrically and adjusted to approximately 58%, which is suitable for solid-state fermentation.

### 2.3. Inoculation and Solid-State Fermentation

PN was propagated on potato dextrose agar (PDA) and incubated aerobically at 25 °C for 7 days to allow sporulation. The yeast strains DH and KM were cultivated on yeast extract–peptone–dextrose (YPD) agar (Sigma-Aldrich, St. Louis, MO, USA) at 30 °C for 3 days under aerobic conditions. LP was grown on de Man–Rogosa–Sharpe (MRS) agar (Sigma-Aldrich, St. Louis, MO, USA) and incubated anaerobically at 37 °C for 3 days using an anaerobic jar equipped with an AnaeroGen pack (Thermo Fisher Scientific, Waltham, MA, USA).

Preparation of microbial inocula followed the protocol previously described in [15]. Briefly, spores of PN were harvested by rinsing the agar surface with phosphate-buffered saline (PBS) and separating spores from mycelial fragments using a cell strainer (Corning Inc., Corning, NY, USA). Spore concentrations were determined using a hemocytometer. For yeast and bacterial cultures, cells were collected using PBS and quantified by measuring optical density at 600 nm (OD600) with a Varioskan^®^ spectrophotometer (Thermo Fisher Scientific, Waltham, MA, USA). Colony-forming units (CFU/mL) were subsequently estimated using strain-specific calibration curves, as described in [15].

The inoculum levels applied in this study were consistent with those previously reported in [15]. The fungal inoculum was adjusted to 1.0 × 10^6^ spores/g of rehydrated soybeans, while yeast and lactic acid bacterial inocula were standardised to 6.0 log CFU/g. These inoculation levels were selected based on preliminary optimisation trials to promote balanced microbial growth and avoid rapid dominance by a single microorganism during co-culture fermentation.

The inoculum was applied to cooled rehydrated soybeans distributed in Petri dishes. In co-culture treatments, all microorganisms were inoculated simultaneously to enable concurrent colonisation and metabolic interaction throughout fermentation. Solid-state fermentation was performed using 50 g of soybean substrate per replicate and carried out in triplicate. Fermentation conditions followed the procedure described in [15], with incubation at 25 °C for 72 h under static conditions. Samples were incubated in covered but non-sealed Petri dishes to permit passive gas exchange. Oxygen concentrations were not directly monitored; therefore, references to oxygen gradients in this study are inferred from fungal surface growth behaviour rather than experimentally measured values.

Uninoculated soybeans subjected to identical incubation conditions served as the control treatment. During fermentation, approximately 5 g of substrate was aseptically sampled each day for microbial enumeration and subsequent chemical analyses.

### 2.4. Microbial Enumeration

Microbial enumeration was performed following the procedure described in [15] with minor modifications. Briefly, 5 g of fermented soybean sample was transferred into a 50 mL Falcon tube and homogenised with 10 mL of 10% PBS using vortex mixing for 1 min. The resulting suspension was subsequently subjected to serial dilution using 10% PBS prior to plating.

For samples fermented with PN, KM, and DH, diluted aliquots were plated onto Rose Bengal Agar (RBA) supplemented with chloramphenicol (Sigma-Aldrich, St. Louis, MO, USA) to inhibit bacterial growth. Samples fermented with LP were plated on MRS agar containing cycloheximide (Sigma-Aldrich, St. Louis, MO, USA) to suppress fungal growth.

Incubation conditions followed those described in [15]. RBA plates were incubated aerobically at 25 °C for 7 days to allow fungal colony development, while yeast plates were incubated at 30 °C for 3 days. MRS plates were incubated anaerobically at 37 °C for 3 days using an anaerobic jar with an AnaeroGen pack (Thermo Fisher Scientific, Waltham, MA, USA). Microbial counts were determined after incubation, with all analyses performed in triplicate.

### 2.5. Sample Preparation for Chemical Analysis

Sample preparation was performed according to the procedure described in [15] with minor modifications. Briefly, 45 g of fermented soybean sample was rapidly frozen using liquid nitrogen for approximately 20 s to halt enzymatic activity. The frozen material was then pulverised for 1 min using a coffee grinder (Rommelsbacher EGK 200, Rommelsbacher, Germany) to obtain a homogeneous powder. The ground samples were subsequently transferred into 50 mL Falcon tubes and stored at −20 °C until further analyses were conducted.

### 2.6. pH Measurement

pH determination was conducted following the procedure described in [15] with slight modifications. Briefly, 0.5 g of the ground soybean sample was transferred into a 5 mL centrifuge tube and mixed with 1 mL of Milli-Q water. The suspension was homogenised by vortex mixing prior to measurement. The pH of each sample was then recorded using a pH meter (LaquaAct, Horiba, Japan). All measurements were performed in triplicate.

### 2.7. High-Performance Liquid Chromatography (HPLC) for Sugar and Organic Acid Analysis

Quantification of fermentation metabolites was performed following the analytical procedure described in [15] with minor modifications. Briefly, 0.5 g of ground soybean sample was transferred into a 5 mL centrifuge tube and extracted with 2 mL of Milli-Q water. The mixture was homogenised by vortex mixing for 1 min and subsequently clarified by centrifugation at 3405× *g* for 10 min. The resulting supernatant was filtered through a 0.22 μm Claristep syringeless filter (Sartorius, Göttingen, Germany) prior to chromatographic analysis.

Metabolite separation and quantification were carried out using an Agilent Infinity 1200 liquid chromatography system (Agilent Technologies, Santa Clara, CA, USA) equipped with both a diode array detector (DAD) and a refractive index detector (RID). Chromatographic separation was achieved using an Aminex HPX-87H column (300 mm × 7.8 mm). The mobile phase consisted of 0.005 M H_2_SO_4_ delivered under isocratic conditions at a flow rate of 0.6 mL min^−1^. The column and RID temperatures were maintained at 35 °C, and the injection volume was set to 20 μL.

Chromatographic data acquisition and integration were performed using Agilent OpenLab CDS ChemStation Edition C.01.10 (Agilent Technologies, Santa Clara, CA, USA). Processed data were exported to Microsoft Excel (Microsoft Office Professional Plus 2016) for further analysis. Quantification was conducted using external calibration with analytical standards of glucose, fructose, sucrose, raffinose, stachyose, lactic acid, acetic acid, citric acid, and ethanol (Sigma-Aldrich, St. Louis, MO, USA). Calibration curves were generated within appropriate concentration ranges for each compound, and metabolite concentrations were calculated based on peak area integration with correlation coefficients (R^2^) ≥ 0.98.

### 2.8. Amino Acid Analysis

Free amino acid (FAA) profiles were determined using an ARACUS Amino Acid Analyzer (MembraPure, Berlin, Germany) following the analytical protocol reported in [15] with minor modifications. Norvaline was used as the internal standard for quantification. Briefly, 0.4 g of ground soybean sample was transferred into a 5 mL centrifuge tube and mixed with 350 µL of 10 mM norvaline solution together with 3650 µL of 0.1 N HCl. The mixture was homogenised using a HulaMixer™ (Thermo Fisher Scientific, Waltham, MA, USA) for 30 min and subsequently centrifuged at 0 °C and 3405× *g* for 10 min.

Following centrifugation, 800 µL of the supernatant was transferred to a 1 mL microcentrifuge tube containing 200 µL of 40% trichloroacetic acid (TCA) to precipitate proteins. The mixture was vortexed for 10 min and then centrifuged at 4 °C and 25,830× *g* for 5 min. A 100 µL aliquot of the clarified supernatant was combined with 600 µL of 0.1 N HCl in an HPLC vial prior to analysis.

Chromatographic separation was performed using a lithium-based column system (MembraPure, Berlin, Germany) consisting of a pre-column and a separation column (150 mm × 4 mm). Eluent solutions (A–F), as well as wash and ninhydrin derivatisation reagents, were supplied by the manufacturer (MembraPure, Berlin, Germany). The eluent flow rate was maintained at 200 µL min^−1^, and the post-column reactor temperature was set to 130 °C. Detection of amino acids was performed spectrophotometrically at 570 nm, with proline monitored at 440 nm. Quantification was carried out using calibration factors provided in the instrument software, as described in [15].

### 2.9. Fatty Acid Methyl Ester (FAME) Analysis via GC-FID

Fatty acid extraction and analysis were performed following the procedure described in [15], which was adapted from the standard method GB 5009.168-2016 [16]. Briefly, lipids from the fermented soybean samples were extracted and converted into fatty acid methyl esters prior to chromatographic analysis.

Fatty acid composition was determined using gas chromatography equipped with a flame ionisation detector (GC-FID; Agilent 7890A, Agilent Technologies, Santa Clara, CA, USA). Individual fatty acids were identified by comparing their retention times with those of reference standards from a Supelco 37-component fatty acid methyl ester mixture. The fatty acid composition of each sample was expressed as the relative percentage of total detected fatty acids or grouped fatty acid classes.

### 2.10. Electronic Tongue (E-Tongue) Taste Profiling

Taste-related properties of the fermented soybean samples were evaluated using an ASTREE electronic tongue system (Alpha MOS, Toulouse, France), following the procedure described in [15] with minor modifications. The system employed seven potentiometric sensors (AHS, ANS, CPS, CTS, NMS, PKS, and SCS) together with an Ag/AgCl reference electrode to record taste responses.

For sample preparation, 0.3 g of ground soybean sample was transferred into a 50 mL Falcon tube and mixed with 30 mL of Milli-Q water. The suspension was first vortexed to ensure complete dispersion and subsequently agitated on a rotary shaker for 30 min to facilitate extraction of soluble taste compounds. The mixture was then centrifuged at 3405× *g* for 30 min, and the clarified supernatant was collected for e-tongue analysis.

Measurements were conducted at room temperature. Prior to sample analysis, the sensors were conditioned according to the manufacturer’s recommendations to stabilise sensor responses. Between measurements, the sensors and reference electrode were rinsed with distilled water for 10 s to prevent cross-contamination between samples.

Each sample was analysed for 120 s, and measurements were repeated nine times to ensure system stability. The sensor response recorded at the 120 s time point from the final six replicates was extracted and averaged to generate the raw sensor signal values used for subsequent data analysis.

### 2.11. Volatile Compound Profiling via GC-MS

For volatile profiling, 0.5 g of sample was weighed into a 20 mL headspace vial followed by the addition of 100 μL of internal standard (2,3-dimethoxytoulene) (1 ppm). The samples were run through GC-MS (GC: Agilent 7890B GC; MS: Agilent 5977B MSD). Headspace solid-phase microextraction (HS-SPME) was used to extract volatile compounds in the samples. The incubation time was 10 min, and the incubation temperature was set at 40 °C. The extraction time was set at 30 min. Volatile profiling was conducted using a DB-WAX UI (30 m × 0.25 mm) column. The inlet temperature was set at 250 °C and the split ratio, at 100:1. The flow rate was set at 1 mL/min. Volatile compounds were semi-quantified relative to the internal standard (2,3-dimethoxytoluene), as authentic external standards were not used in this study. Compound identification was based on mass spectral matching against an in-house library (minimum match score ≥ 80%) combined with retention index agreement within ±20 units. Only compounds meeting both criteria were reported. The amount of aroma compounds in the samples was normalised to the internal standard aliquoted into each of the samples prior to GC analysis.

### 2.12. Calculation of Odour Activity Values (OAVs)

Odour activity values (OAVs) were calculated to estimate the relative contribution of individual volatile compounds to the overall aroma profile. Compound concentrations µg/g wet weight) were obtained from GC-MS analysis as described above. Reported odour thresholds for each compound were obtained from the literature and are expressed in the same units (µg/g) [17]. The OAV of each volatile was determined using the following equation:

$$\mathrm{OAV}\;=\;\frac{T_{i}}{C_{i}}$$

where *C_i_* is the measured concentration of compound *I* (µg/g) and *T_i_* is the published odour threshold of the same compound (µg/g). Compounds with OAV > 1 were considered aroma-active and likely to influence the sensory characteristics of the fermented samples, while compounds with OAV < 1 were considered to have negligible individual impact. All calculations were performed in Microsoft Excel 365 (Microsoft Corporation, Redmond, WA, USA, 2018) [18], and the results were tabulated for further interpretation.

### 2.13. Statistical Analysis

The experiments were conducted as three independent biological fermentation batches prepared on separate days, each with independently prepared soybean substrate and inoculum. Data are presented as mean ± standard deviation (SD). Prior to performing analysis of variance (ANOVA), normality of residuals was assessed using the Shapiro–Wilk test and homogeneity of variance was evaluated using Levene’s test. One-way ANOVA followed by Tukey’s HSD post hoc test was used to determine significant differences among treatments (*p* < 0.05). Statistical analyses were performed in R (v4.x) using the base stats package and the agricolae package. Principal component analysis (PCA) was also conducted in R [19].

## 3. Results and Discussion

### 3.1. Changes in Microbial Population During Fermentation

**Figure 1:**
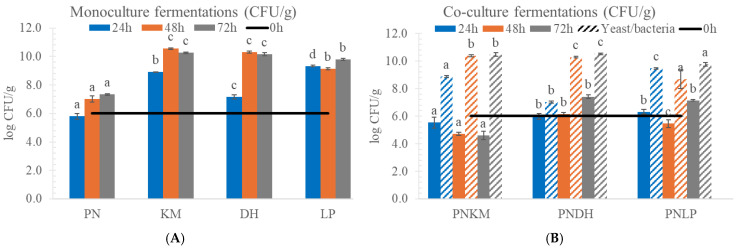
Microbial populations during solid-state fermentation (SSF) of soybean over 72 h. (**A**) Monoculture fermentations. (**B**) Co-culture fermentations. PN = *Penicillium nalgiovense*; KM = *Kluyveromyces marxianus*; DH = *Debaryomyces hansenii*; LP = *Lactiplantibacillus plantarum*. The black line represents the initial inoculum concentration. Solid bars indicate PN populations and shaded bars indicate co-culture partner populations. Different letters indicate statistically significant differences among treatments (ANOVA with Tukey’s HSD, *p* < 0.05). Solid-colour bars = *Penicillium nalgiovense* (PN); shaded bars = symbiotic bacteria/yeast partner (KM, DH, LP, depending on the co-culture).

Soybeans, which contain substantial amounts of protein, lipids, and carbohydrates, provide a nutrient base that readily supports microbial growth [20]. When the different strains were introduced into this matrix, each followed its own growth pattern over the 72 h period. Most cultures increased steadily in cell counts; the single exception occurred in the PNKM treatment, where PN exhibited reduced visible surface colonisation, consistent with its comparatively lower plant counts in this treatment (Figure 1). Earlier studies suggest that KM can generate volatile metabolites that suppress the growth of Penicillium species [21], which offers a plausible explanation for the lack of visible PN mycelium. Even so, the absence of surface colonisation does not necessarily mean that PN was inactive. Filamentous fungi can secrete enzymes without producing high numbers of detectable spores, so PN may still have influenced the biochemical changes occurring in this co-culture.

In contrast, PN grew vigorously in all other treatments, reaching high population levels and exhibiting extensive surface colonisation by the end of fermentation. This is consistent with its known affinity for oxygenated, protein- and lipid-rich substrates [22]. The two yeasts exhibited contrasting behaviour: KM multiplied rapidly during the first day, likely drawing on the simple sugars generated during the heat treatment of the soybeans [23], whereas DH increased more slowly, which is characteristic of its adaptation to salty or fatty environments rather than legumes [24]. The lactic acid bacterium LP displayed a steady rise throughout the process, reaching its highest population around 48 h, reflecting its ability to grow even as the environment became more acidic and increasingly reduced due to its own metabolism.

In addition, targeted LC-MS screening confirmed the absence of major Penicillium-associated mycotoxins, including patulin and aspergillic acid derivates (Table S5), under the fermentation conditions applied. These findings support the strain-level safety of PN in this soybean fermentation system.

### 3.2. Substrate Utilisation and Metabolite Production

**Figure 2:**
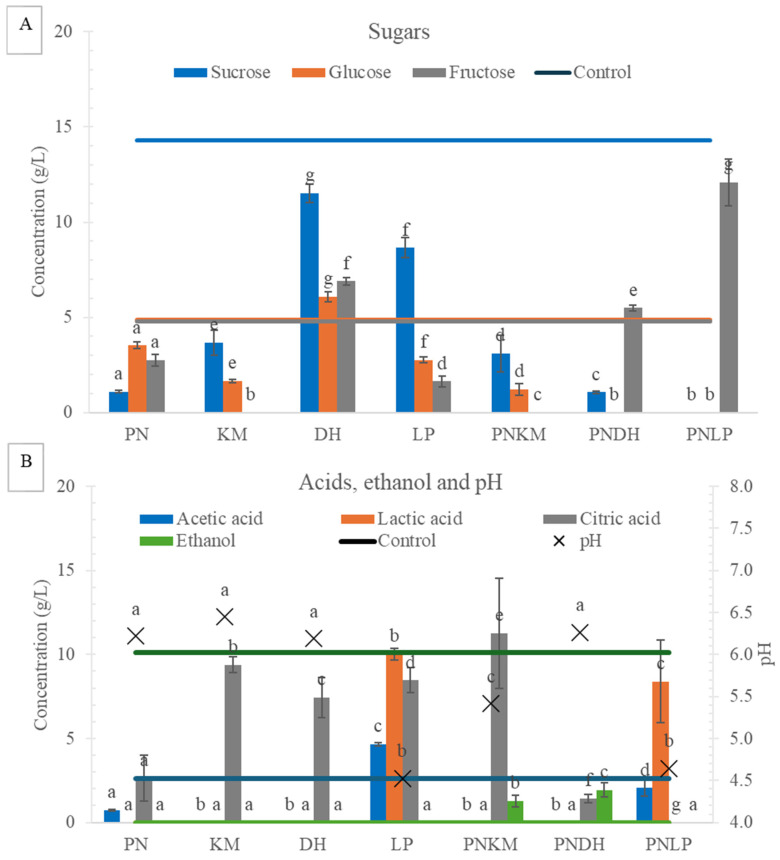
Substrate utilisation and metabolite production after 72 h of soybean fermentation. (**A**) Residual sugar concentrations. (**B**) Organic acids and ethanol concentrations. PN = *Penicillium nalgiovense*; KM = *Kluyveromyces marxianus*; DH = *Debaryomyces hansenii*; LP = *Lactiplantibacillus plantarum*. The control represents uninoculated soybean. Different letters indicate statistically significant differences among treatments (ANOVA with Tukey’s HSD, *p* < 0.05).

Clear and strain-dependent differences in carbohydrate utilisation emerged after 72 h of fermentation (Figure 2). All fermented samples showed a marked reduction in total sugars relative to the uninoculated control, except for those inoculated with DH. This pattern aligns with the yeast’s semi-fermentative-to-non-fermentative metabolic strategy, in which available carbohydrates are channelled predominantly through respiratory pathways rather than converted into typical fermentative end-products [24].

A markedly different outcome was observed in the PNDH co-culture when compared with the DH monoculture. In the mixed fermentation, glucose was almost fully depleted by 72 h, indicating that PN’s enzymatic degradation of soybean macromolecules generated additional accessible substrates for DH. PN is well documented for its secretion of diverse extracellular hydrolases capable of releasing simple sugars, amino acids, and free fatty acids from complex plant matrices. This enhanced availability of low-molecular-weight substrates likely underpins the pronounced carbohydrate utilisation seen in the co-culture relative to DH alone.

Sucrose metabolism further demonstrated strain-specific behaviour. LP by itself retained considerable sucrose, whereas co-fermentation with PN resulted in both substantial sucrose and glucose depletion and a concurrent increase in fructose concentration. The rise in fructose aligns with invertase activity reported in various Penicillium species, which catalyses the cleavage of sucrose into glucose and fructose [25]. The acidic conditions generated by LP likely enhanced PN-associated invertase activity, as this enzyme typically performs optimally under moderately acidic conditions [26]. The more extensive sucrose degradation in the PNLP treatment therefore suggests a synergistic pH–enzyme relationship in which LP modifies the environment in a manner that strengthens PN’s hydrolytic activity.

Organic acid profiles further differentiated the fermentation systems. Treatments containing LP produced substantial lactic acid, consistent with its homofermentative metabolic profile [27]. Citrate utilisation, however, varied considerably between strains. The most pronounced citrate depletion occurred in PN-containing fermentations, with PNLP exhibiting near-complete citrate removal. Both PN and some LAB strains are known to harbour citrate lyase pathways, although enzyme activity is typically enhanced under low-oxygen conditions. This is coherent with fungal citrate lyase behaviour, which contributes acetyl-CoA for fatty acid synthesis [28]. The dense PN mycelium layer may have reduced oxygen penetration into the substrate, potentially generating localised microzones that could favour citrate metabolism [29]. In contrast, citrate utilisation in LP monocultures remained limited, implying that in the absence of PN’s surface growth, LP’s citrate-degrading activity remained limited, suggesting that microenvironmental differences may influence pathway activation.

Small quantities of ethanol were detected only in PNKM and PNDH, but not in the corresponding monocultures. KM typically produces ethanol under oxygen-limited conditions [30], and the presence of ethanol in the PNKM co-culture is consistent with reduced oxygen availability associated with PN’s surface growth, although oxygen levels were not directly measured. DH does not normally produce ethanol aerobically [31], yet PNDH contained trace ethanol, again suggesting that PN’s surface growth may have contributed to localised oxygen limitation, which could activate fermentative pathways [32]. These findings indicate that PN does more than release substrates. It also restructures microenvironmental conditions in ways that alter the metabolic behaviour of accompanying microbes.

### 3.3. Free Amino Acid and E-Tongue Profiles

**Figure 3:**
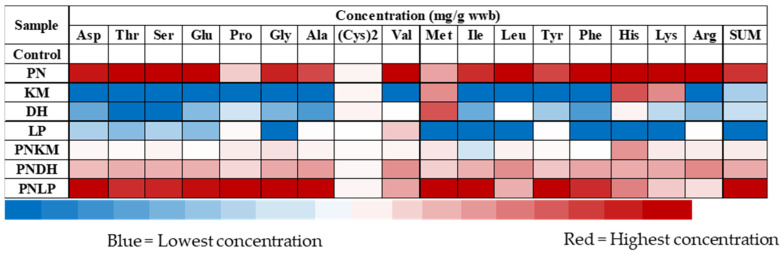
Heatmap showing the relative abundance of free amino acids in soybean samples after 72 h of fermentation. Rows represent individual amino acids and columns represent treatment groups. PN = *Penicillium nalgiovense*; KM = *Kluyveromyces marxianus*; DH = *Debaryomyces hansenii*; LP = *Lactiplantibacillus plantarum*. Colour intensity reflects relative concentration differences compared to the uninoculated control, with red indicating higher relative abundance and blue indicating lower relative abundance. As each amino acid has a distinct concentration range, the colour scale represents relative variation within each amino acid rather than absolute values on a shared numeric scale. Quantitative concentration data (mg/g wet-weight basis) are provided in Table S1.

The fermentation treatments produced pronounced differences in free amino acid (FAA) accumulation, reflecting each microorganism’s proteolytic capacity and its ability to access soybean proteins (Figure 3). The most pronounced increases in free amino acids (FAAs) occurred in fermentations that included PN, whether applied on its own or together with other microorganisms. PN’s broad protease and peptidase repertoire—capable of hydrolysing key soybean storage proteins such as glycinin and β-conglycinin [33]—is the most plausible driver of the substantial FAA accumulation relative to the unfermented control. In contrast, the KM, DH, and LP monocultures produced FAA levels comparable to the control, indicating minimal proteolytic action by these strains when grown individually on soybean.

Among all treatments, the PNLP co-culture yielded the highest total FAA concentration. This outcome reflects the complementary metabolic capacities of the two organisms: PN-containing fermentations showed significantly higher free amino acid accumulation, indicating enhanced proteolysis. Based on literature reports, several PN proteases, including cathepsin L and B, are known to exhibit optimal activity under mildly acidic conditions [34]. The increased FAA levels in PNLP may reflect pH-dependent enhancement of PN proteases through LP-mediated acidification of proteolytic activity. However, enzyme activity was not directly measured in this study.

Interestingly, FAA concentrations in PN–yeast co-cultures were lower than in the PN monoculture despite PN’s strong protease output. This suggests that KM and DH actively metabolised the FAAs liberated by PN, either to support their nitrogen requirements or to generate volatile compounds through amino acid catabolism. These findings emphasise that co-culture systems do not simply amplify proteolysis; rather, microbial interactions determine how liberated nitrogen is partitioned between growth and aroma-forming pathways.

**Figure 4:**
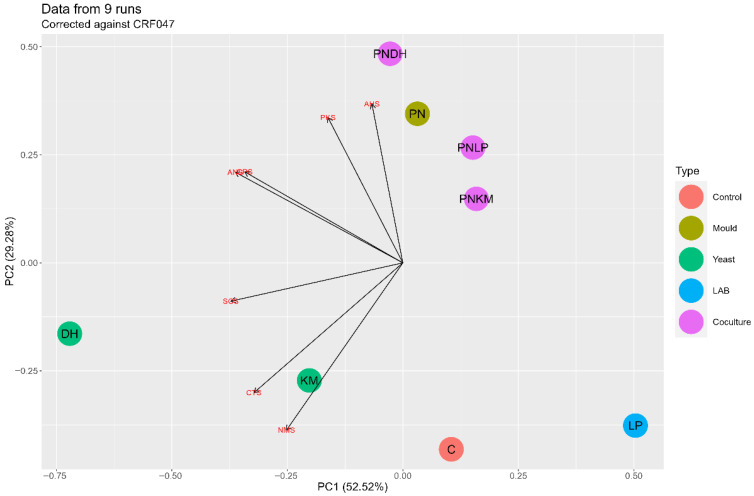
Principal component analysis (PCA) of electronic tongue (e-tongue) responses for fermented soybean samples after 72 h. PN = *Penicillium nalgiovense*; KM = *Kluyveromyces marxianus*; DH = *Debaryomyces hansenii*; LP = *Lactiplantibacillus plantarum*. The control represents uninoculated soybean.

**Figure 5:**
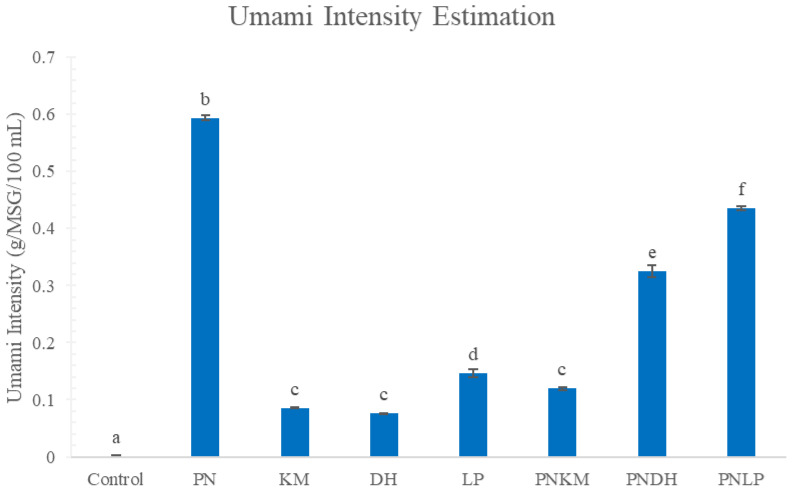
Predicted umami intensity of fermented soybean samples after 72 h as measured by electronic tongue (e-tongue) analysis. PN = *Penicillium nalgiovense*; KM = *Kluyveromyces marxianus*; DH = *Debaryomyces hansenii*; LP = *Lactiplantibacillus plantarum*. Different letters indicate statistically significant differences among treatments (ANOVA with Tukey’s HSD, *p* < 0.05).

**Figure 6:**
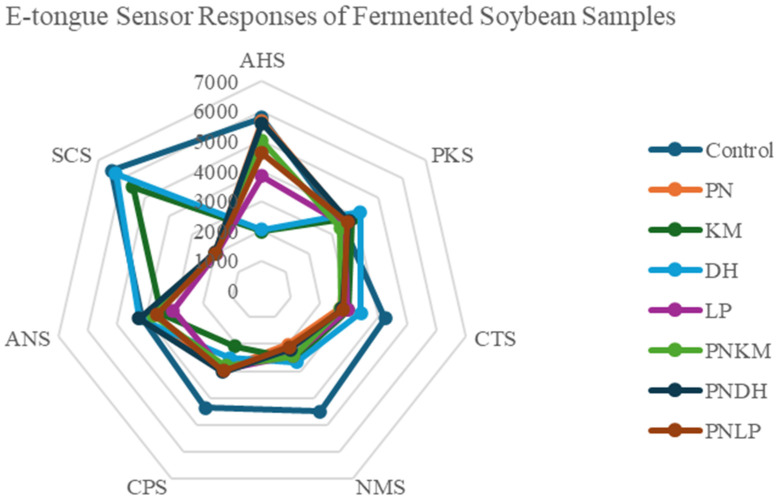
Electronic tongue (e-tongue) radar plot showing sensor response patterns across seven taste attributes. PN = *Penicillium nalgiovense*; KM = *Kluyveromyces marxianus*; DH = *Debaryomyces hansenii*; LP = *Lactiplantibacillus plantarum*. AHS = astringency; PKS = pungency; CTS = saltiness; NMS = umami; CPS = sourness; ANS = bitterness; SCS = aftertaste bitterness.

Sensory analyses provided further context for these biochemical changes. Electronic tongue data (Figure 4) showed that PN-containing fermentations formed a distinct cluster relative to the other treatments. Umami intensity measurements (Figure 5) were also highest in PN-based fermentations, consistent with their elevated levels of glutamate, aspartate, and other umami-associated FAAs (Table 1). Because FAAs are well-established contributors to umami perception, their abundance in the PN monoculture exhibited a stronger predicted umami response based on electronic tongue measurements. Radar profiles from the electronic tongue (Figure 6) further illustrate this pattern, with PN producing a notably stronger umami signal than the other strains.

The PNLP co-culture also exhibited significant umami intensity, second only to PN alone, suggesting that additional mechanisms beyond FAA accumulation shape its sensory profile. LP monocultures generated measurable umami despite minimal FAA production, implying a contribution from umami-active peptides. LAB such as LP are known to express diverse proteolytic enzymes—including endopeptidases, carboxypeptidases, and exopeptidases—that release short peptides with pronounced umami and kokumi characteristics [35]. In the PNLP system, these LAB-derived peptides likely acted synergistically with PN-driven FAA generation, producing a more layered set of umami-active compounds. This combined effect mirrors observations in fermented meat systems, where PN–LAB interactions are reported to enrich flavour, enhance palatability, and strengthen microbial stability through complementary biochemical pathways [36].

### 3.4. Fatty Acid Profile

**Figure 7:**
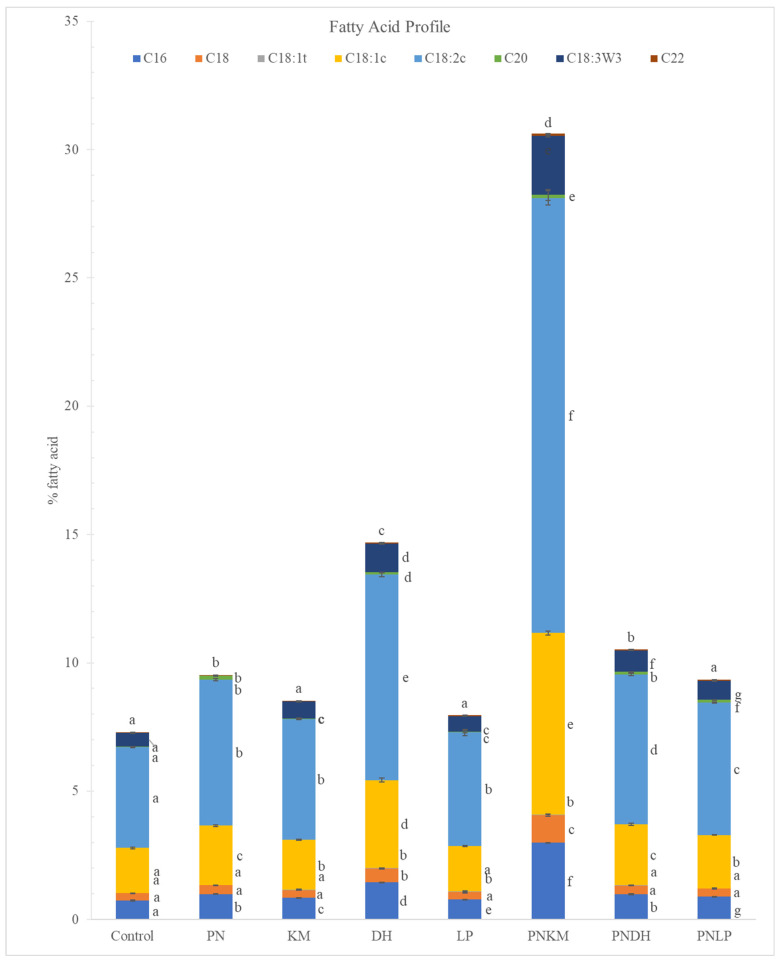
Fatty acid profile of fermented soybean samples after 72 h. PN = *Penicillium nalgiovense*; KM = *Kluyveromyces marxianus*; DH = *Debaryomyces hansenii*; LP = *Lactiplantibacillus plantarum*. The control represents uninoculated soybean. Different letters indicate statistically significant differences among treatments (ANOVA with Tukey’s HSD, *p* < 0.05).

Soybean oil contains a substantial proportion of polyunsaturated lipids, with 10% palmitic acid (C16:0), 4% stearic acid (C18:0), 23% oleic acid (C18:1), 51% linoleic acid (C18:2), and 7–10% α-linolenic acid (C18:3) [37]. These lipids serve as important precursors for many aroma-relevant molecules formed during fermentation. The detailed fatty acid composition of the fermented soybean samples is presented in Table S2 in the Supplementary Materials. In this study, most microbial treatments resulted in higher free fatty acid levels compared with the unfermented control (Figure 7), indicating that lipid hydrolysis occurred during fermentation. KM and LP were the main exceptions, showing little deviation from the baseline, which is consistent with their modest endogenous lipase activity and limited ability to liberate fatty acids from triacylglycerols. Across all fermentations, the PNKM combination stood out for the increase in total fatty acids, and linoleic acid (C18:2) rose more than any other lipid measured. This pattern implies that PN was actively breaking down lipids in this setting, and its activity may have been influenced by how KM altered the fermentation environment. Linoleic acid is important here because it feeds into a range of volatile pathways; once it undergoes oxidation, it can give rise to aldehydes such as (E,E)-2,4-decadienal, a compound often associated with “oily” or “chicken-like” aromas [38].

A similar trend was observed for α-linolenic acid (C18:3), which also increased substantially in PNKM. Like linoleic acid, α-linolenic acid is highly susceptible to enzymatic and autoxidative degradation, generating important aroma volatiles such as nonanal, (E,E)-2,4-decadienal, and 1-octen-3-ol, compounds that have characteristic aromas of Beijing roasted duck and other roasted or fermented foods [39]. The increase in polyunsaturated fatty acids is also consistent with the metabolic capabilities of *Penicillium* spp., which can synthesise α-linolenic acid through a sequential desaturation pathway, stearate → oleate → linoleate → linoleate, using acetate as a precursor [40]. This ability not only contributes to the pool of free fatty acids available for aroma formation but also enhances the nutritional profile of the fermented soybean product, given the dietary relevance of polyunsaturated fatty acids.

### 3.5. Volatile Profile

**Figure 8:**
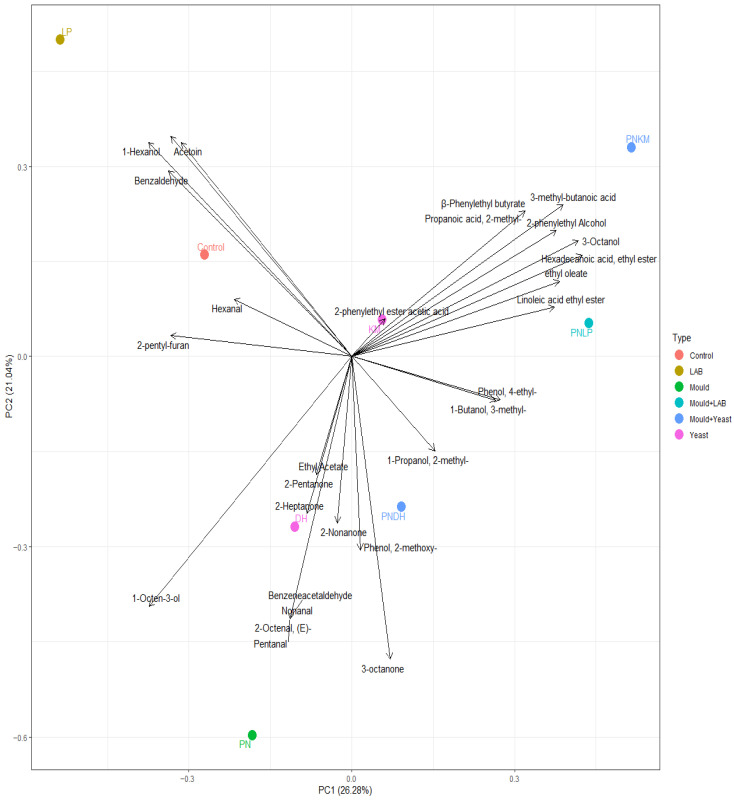
Principal component analysis (PCA) of volatile compound profiles in fermented soybean samples after 72 h. PN = *Penicillium nalgiovense*; KM = *Kluyveromyces marxianus*; DH = *Debaryomyces hansenii*; LP = *Lactiplantibacillus plantarum*. The control represents uninoculated soybean.

The PCA results for the volatile dataset (Figure 8) showed a clear divide between the unfermented soybeans and all fermented samples, demonstrating how strongly microbial activity reshaped the aromatic landscape. Prior to fermentation, the volatile profile was dominated by compounds such as hexanal and 2-pentylfuran (Table S3), which are the main contributors to the characteristic “beany” or “green” notes of raw soy. These molecules have very low odour thresholds and thus exert a disproportionate influence on sensory perception [5]. Following fermentation, their concentrations dropped sharply in every treatment. This is consistent with the ability of moulds, yeasts, and LAB to transform reactive aldehydes either through reduction or by channelling them into alternative pathways, thereby reducing their sensory prominence [41]. The removal of these key off-flavour compounds suggests a potential improvement in the underlying aromatic quality of soybeans.

Although all fermented samples differed from the unfermented substrate, each microbial treatment produced its own aroma signature. OAV analysis helped identify the compounds that contributed most strongly to these differences. The calculated odour activity values (OAVs) for all detected volatile compounds are summarised in Table S4 in the Supplementary Materials. In fermentations inoculated only with PN, 1-octen-3-ol emerged as a defining feature. This molecule, produced via fungal oxidation of linoleic acid, is associated with the familiar mushroom-like aroma characteristic of mould-based fermentations. Its presence sharply contrasted with certain co-cultures—most notably PNKM—where 1-octen-3-ol was markedly reduced or absent. Instead of fungal oxidation products, these co-cultures accumulated fatty acid ethyl esters, implying that the metabolic flow had shifted toward esterification. Ethyl linoleate, present in both PNKM and PNLP, imparts fruity, fatty, and waxy notes and likely formed through the reaction of linoleic acid with ethanol generated by KM under restricted oxygen conditions. PNKM also contained ethyl palmitate, a compound with creamy and buttery attributes. The altered fatty acid and ester profiles observed in the PN-yeast co-cultures suggest potential shifts in lipid metabolism compared to monocultures.

Compounds associated with sweet or floral aromatics varied in a similarly treatment-specific manner. PNKM contained substantial amounts of 2-phenylethyl alcohol and its ester, 2-phenylethyl acetate—both known for their rose-like and honey-sweet notes. KM monocultures produced these volatiles as well, reflecting KM’s ability to catabolise phenylalanine via transamination and decarboxylation [23]. The detection of β-phenylethyl butyrate uniquely in PNKM suggests esterification mediated by PN-derived acyltransferases [42], with ethanol availability (Figure 2B) likely facilitating this chemistry.

Several aroma attributes associated with dairy-like, acidic, or “cheesy” characteristics also showed strong strain-specific origins. PNKM contained notable amounts of 3-methylbutanoic and 2-methylpropanoic acids, both branched-chain fatty acids derived from amino acid catabolism. LP monocultures, by contrast, accumulated acetoin, a buttery and creamy-smelling compound linked with citrate metabolism. Some monocultures also produced hexanoic acid; however, this compound was absent in PNLP, where 3-methylbutanoic acid prevailed, suggesting that different oxidative pathways were favoured—potentially via oxidation of 3-methyl-1-butanol [43].

One of the clearest sensory markers distinguishing the PNKM and PNLP treatments was their elevated levels of 4-ethylphenol, which imparts smoky, meaty, and ham-like aromas. This compound results from decarboxylation of phenylalanine or p-coumaric acid [44]. The co-cultures likely promoted its formation by combining PN’s strong proteolysis (which increases precursor availability) with decarboxylase activities from KM or LP. Additional alcohols derived from yeast metabolism, including 2-methyl-1-propanol and 3-methyl-1-butanol, were abundant in PNDH, lending fermented, malty nuances to that treatment. Although DH monocultures did not produce 2-phenylethyl alcohol, its appearance in PNDH suggests that PN’s protein degradation released phenylalanine in sufficient amounts for DH to access this pathway [45].

It should be noted that these interpretations are based on chemical quantification and OAV estimation, and sensory validation through trained panel evaluation will be necessary to confirm perceptible flavour changes.

## 4. Limitations and Future Work

Although the findings here point to PN as a promising partner organism for shaping the flavour, taste, and aroma of fermented soybeans, the study also has clear limitations that open avenues for further work. One important limitation is that only a single PN strain was examined, and the microbial partners—both LAB and yeasts—were limited to a small set of combinations. Given the substantial strain-to-strain variability documented in PN and LAB, future studies should examine a broader panel of isolates, including strains with differing proteolytic, lipolytic, and esterification capacities, to determine whether the observed synergies generalise across genotypes. Second, the current study relied on 72 h solid-state fermentation under static, laboratory-controlled conditions. Fermentation dynamics, particularly oxygen availability, moisture gradients, and metabolite diffusion, are known to shift significantly at pilot and industrial scales. Future work should therefore incorporate controlled bioreactors or scaled SSF systems to validate whether the metabolic interactions seen here remain stable under more heterogenous conditions.

Another limitation is that the volatile and taste analyses were largely chemical- and instrument-based. Although electronic tongue analysis and odour activity value (OAV) calculations provided mechanistic insight into taste and aroma potential, no trained sensory panel or consumer validation was conducted in this study. Therefore, the reported sensory changes represent predicted sensory modulation based on instrumental measurements rather than confirmed human perception. Follow-up trials incorporating trained sensory panels, consumer acceptance tests, and descriptive profiling would be essential to validate whether the aroma and umami enhancements observed analytically translate into perceptible and desirable sensory improvements. Additionally, this study emphasised proteolysis, lipolysis, and volatile generation, but did not characterise the transcriptomic or proteomic basis of these interactions. Multi-omics approaches, including meta transcriptomics, targeted proteomics, and metabolite flux analysis, would help understand which metabolic pathways are activated in specific co-cultures, especially in PNKM and PNLP where metabolic redirection was prominent.

## 5. Conclusions

The findings of this work demonstrate that PN serves as a major driver of both biochemical and sensory changes in fermented soybean systems. PN exerted a much stronger influence on the soybean fermentations than any of the yeast or LAB strains used on their own. Its extensive protease and lipase activities, together with the way it modifies its surroundings during growth, resulted in far more pronounced biochemical shifts. In the PN-only fermentation, the mould generated noticeably higher amounts of savoury free amino acids along with several key aroma compounds. When PN was cultivated together with LP, KM, or DH, each pairing took on a different chemical direction; the sets of metabolites were not only different in kind but also in how strongly they accumulated. The PNLP and PNKM combinations, for instance, produced more esters, floral alcohols, savoury volatiles, and a broader range of polyunsaturated fatty acids than any of the monocultures. These shifts suggest that once PN is present, the fermentation environment changes enough for the partner microbes to tap into metabolic options they do not usually activate on their own. PN-based fermentations consistently reduced key aldehydes associated with beany off-flavour descriptors.

What emerges from this work is that PN can be used intentionally as a functional starter for soybean fermentations. Although it is a familiar organism in the context of dry-cured meat production, its behaviour on plant substrates has not been deeply characterised. In this system, PN altered how accessible different parts of the substrate were, may have influenced surface oxygen exposure during solid-state fermentation, and seemed to create conditions that encouraged other microbes to express metabolic traits they normally would not. These observations suggest the potential for designing PN-based culture combinations to modulate flavour-related chemical profiles in soybean substrates. PN-driven mixed fermentations could offer a practical route to overcoming long-standing sensory limitations of soy and to developing more appealing fermented ingredients for plant-based applications.

## Figures and Tables

**Table 1:** Umami-relevant free amino acids in fermented soybean samples after 72 h. Values are expressed as mg/g wwb (wet-weight basis). PN = *Penicillium nalgiovense*; KM = *Kluyveromyces marxianus*; DH = *Debaryomyces hansenii*; LP = *Lactiplantibacillus plantarum*. The control represents uninoculated soybean. Different letters indicate statistically significant differences among treatments (ANOVA with Tukey’s HSD, *p* < 0.05). ND = not detected.

Sample	Concentration (mg/g wwb)				
	Glutamic Acid (Glu)	Aspartic Acid (Asp)	Methionine (Met)	Cysteine (Cys)	Total (Glu + Asp + Met + Cys)
Control	0.17 ± 0.04 ^c^	0.07 ± 0.00 ^c^	0.50 ± 0.00 ^b^	ND ^a^	0.74
PN	5.06 ± 0.74 ^g^	1.47 ± 0.27 ^f^	0.82 ± 0.04 ^d^	0.10 ± 0.01 ^b^	7.45
KM	ND ^a^	ND ^a^	0.89 ± 0.22 ^d^	0.09 ± 0.02 ^b^	0.98
DH	0.09 ± 0.11 ^b^	0.03 ± 0.01 ^b^	1.08 ± 0.01 ^a^	0.10 ± 0.03 ^b^	1.30
LP	0.09 ± 0.00 ^b^	0.04 ± 0.01 ^b^	ND ^c^	ND ^a^	0.13
PNKM	0.23 ± 0.08 ^d^	0.12 ± 0.02 ^d^	0.59 ± 0.06 ^b^	0.05 ± 0.03 ^c^	0.99
PNDH	1.71 ± 0.13 ^e^	0.48 ± 0.06 ^e^	0.66 ± 0.04 ^e^	0.08 ± 0.01 ^b^	2.93
PNLP	4.83 ± 0.73 ^f^	1.59 ± 0.23 ^g^	1.36 ± 0.04 ^f^	0.09 ± 0.01 ^b^	7.87

## Data Availability

The data presented in this study are available within the article and the Supplementary Materials. Further inquiries can be directed to the corresponding authors.

## Supplementary Materials

The following supporting information can be downloaded at https://www.mdpi.com/article/10.3390/foods15061053/s1. Table S1: Complete free amino acid concentration data (mg/g wet-weight basis) for fermented soybean samples after 72 h. PN = *Penicillium nalgiovense*; KM = *Kluyveromyces marxianus*; DH = *Debaryomyces hansenii*; LP = *Lactiplantibacillus plantarum*. The control represents uninoculated soybean. Values are presented as mean ± standard deviation (n = 3 biological replicates). Table S2: Fatty acid composition (% of total fatty acids) of fermented soybean samples after 72 h. PN = *Penicillium nalgiovense*; KM = *Kluyveromyces marxianus*; DH = *Debaryomyces hansenii*; LP = *Lactiplantibacillus plantarum*. Values are mean ± standard deviation (n = 3 biological replicates). Table S3: Volatile compounds identified in fermented soybean samples by GC–MS analysis. Volatile compounds identified by GC–MS analysis after 72 h of fermentation. All compounds were semi-quantified relative to the internal standard. Identification was accepted when both mass spectral matching (≥80%) and retention index agreement (±20 units) were achieved. ND = not detected. PN = *Penicillium nalgiovense*; KM = *Kluyveromyces marxianus*; DH = *Debaryomyces hansenii*; LP = *Lactiplantibacillus plantarum*. ND = not detected. Table S4: Odour activity values (OAVs) of identified volatile compounds in fermented soybean samples after 72 h. OAV = odour activity value (calculated as compound concentration divided by odour threshold). PN = *Penicillium nalgiovense*; KM = *Kluyveromyces marxianus*; DH = *Debaryomyces hansenii*; LP = *Lactiplantibacillus plantarum*. Compounds with OAV > 1 are considered potentially aroma-active. Table S5: Targeted LC–MS screening of selected Penicillium-associated secondary metabolites in *Penicillium nalgiovense* (PN) monoculture fermentation after 72 h. Compounds screened included hydroxy-aspergillic acid, neohydroxy-aspergillic acid, neoaspergillic acid, aspergillic acid, and patulin (putative). ND = not detected. Results are shown for biological replicates.
