# Supplementary material for: From Charcuterie to Plant-Based: Harnessing Penicillium nalgiovense for Innovative Soybean Co-Culture Fermentation
Source: Foods. 2026 Mar 17;15(6):1053. doi: 10.3390/foods15061053 (PMC13025917; doi:10.3390/foods15061053)
Supplement: Supplementary file 1 [file foods-15-01053-s001.zip › foods-4147512-supplementary.pdf]

From Charcuterie to Plant-Based: Harnessing *Penicillium nalgiovense* for Innovative Soybean Co-Culture Fermentation

Xin Hui Chin [1,2], Ryan Soh [1], Geraldine Chan [1], Pnelope Ng [1], Aaron Thong [1], Hosam Elhalis [3], Yoganathan Kanagasundaram [1], Yvonne Chow [1,*] and Shao Quan Liu [2,*]

[1] Singapore Institute of Food and Biotechnology Innovation, Agency for Science, Technology and Research, 31 Biopolis Way, Nanos, Singapore 138669, Singapore; chin_xin_hui@a-star.edu.sg (X.H.C.)
[2] Department of Food Science and Technology, Faculty of Science, National University of Singapore, 2 Science Drive 2, Singapore 117543, Singapore
[3] Sydney Technical Centre, AB Mauri, 1 Richardson Place, North Ryde, NSW 2113, Australia
* Correspondence: yvonne_chow@a-star.edu.sg (Y.C.); fstlsq@nus.edu.sg (S.Q.L.)

Table S1: Complete free amino acid concentration data (mg/g wet weight basis) for fermented soybean samples after 72 h. PN = Penicillium nalgiovense; KM = Kluyveromyces marxianus; DH = Debaryomyces hansenii; LP = Lactiplantibacillus plantarum. The control represents uninoculated soybean. Values are presented as mean ± standard deviation (n = 3 biological replicates).

**Sample**	**Amino acid concentrations (mg/g wwb)**																
	**Asp**	**Thr**	**Ser**	**Glu**	**Pro**	**Gly**	**Ala**	**(Cys)2**	**Val**	**Met**	**Ile**	**Leu**	**Tyr**	**Phe**	**His**	**Lys**	**Arg**
Control	0.07 ± 0.00	0.11 ± 0.00	0.05 ± 0.00	0.17 ± 0.04	1.07 ± 0.37	0.05 ± 0.01	0.08 ± 0.01	ND	0.05 ± 0.02	0.50 ± 0.00	0.06 ± 0.07	0.03 ± 0.01	0.08 ± 0.01	0.13 ± 0.09	0.05 ± 0.01	0.06 ± 0.00	0.20 ± 0.03
PN	1.47 ± 0.27	0.91 ± 0.06	1.59 ± 0.15	5.06 ± 0.74	3.90 ± 0.35	0.44 ± 0.05	1.56 ± 0.05	0.10 ± 0.01	1.22 ± 0.11	0.82 ± 0.04	1.00 ± 0.12	1.98 ± 0.18	1.05 ± 0.01	1.81 ± 0.18	1.07 ± 0.13	1.85 ± 0.20	2.11 ± 0.31
KM	ND	ND	ND	ND	ND	ND	ND	0.09 ± 0.02	0.00	0.89 ± 0.22	ND	ND	ND	ND	0.75 ± 0.48	0.89 ± 0.73	ND

DH	0.03 ± 0.01	ND	ND	0.09 ± 0.11	0.87 ± 0.22	0.03 ± 0.01	0.02 ± 0.01	0.10 ± 0.03	0.05 ± 0.01	1.08 ± 0.01	0.03 ± 0.01	0.03 ± 0.02	0.05 ± 0.01	ND	0.10 ± 0.04	0.04 ± 0.02	0.10 ± 0.07
LP	0.04 ± 0.01	0.06 ± 0.01	0.04 ± 0.02	0.09 ± 0.00	1.43 ± 0.20	ND	0.08 ± 0.02	ND	0.30 ± 0.16	ND	ND	ND	0.09 ± 0.02	ND	ND	ND	0.22 ± 0.02
PN KM	0.12 ± 0.02	0.13 ± 0.06	0.12 ± 0.04	0.23 ± 0.08	2.15 ± 0.27	0.11 ± 0.04	0.19 ± 0.16	0.05 ± 0.03	0.10 ± 0.05	0.59 ± 0.06	0.05 ± 0.02	0.14 ± 0.03	0.11 ± 0.05	0.14 ± 0.04	0.49 ± 0.17	0.22 ± 0.06	0.35 ± 0.20
PN DH	0.48 ± 0.06	0.37 ± 0.01	0.53 ± 0.04	1.71 ± 0.13	3.22 ± 0.13	0.20 ± 0.03	0.88 ± 0.05	0.08 ± 0.01	0.57 ± 0.04	0.66 ± 0.04	0.42 ± 0.04	0.90 ± 0.10	0.38 ± 0.05	0.72 ± 0.08	0.39 ± 0.05	0.67 ± 0.07	1.08 ± 0.08
PN LP	1.59 ± 0.23	0.77 ± 0.08	1.38 ± 0.04	4.83 ± 0.73	14.73 ± 2.82	0.49 ± 0.06	2.15 ± 0.16	0.09 ± 0.01	0.47 ± 0.03	1.36 ± 0.04	1.20 ± 0.10	0.65 ± 0.03	1.40 ± 0.10	1.53 ± 0.05	0.56 ± 0.01	0.44 ± 0.03	0.46 ± 0.03

Table S2: Fatty acid composition (% of total fatty acids) of fermented soybean samples after 72 h. PN = Penicillium nalgiovense; KM = Kluyveromyces marxianus; DH = Debaryomyces hansenii; LP = Lactiplantibacillus plantarum. Values are mean ± standard deviation (n = 3 biological replicates).

**Fatty Acid Methyl Ester**		**Control**	**PN**	**KM**	**DH**	**LP**	**PNKM**	**PNDH**	**PNLP**
Myristic Acid	C14	0.00	0.00	0.01 ± 0.00	0.01 ± 0.00	0.01 ± 0.00	0.02 ± 0.00	0.00	0.00
Palmitic Acid	C16	0.74 ± 0.02	1.00 ± 0.01	0.85 ± 0.01	1.45 ± 0.01	0.79 ± 0.00	3.00 ± 0.00	0.98 ± 0.03	0.90 ± 0.00
Palmitoleic Acid	C16:1W7	0.01 ± 0.00	0.01 ± 0.00	0.01 ± 0.00	0.02 ± 0.00	0.01 ± 0.00	0.04 ± 0.00	0.01 ± 0.00	0.01 ± 0.00
Margaric Acid	C17	0.00	0.01 ± 0.00	0.01 ± 0.00	0.01 ± 0.00	0.01 ± 0.00	0.02 ± 0.00	0.01 ± 0.00	0.01 ± 0.00
Magaroleic Acid	C17:1W7	0.00	0.00	0.00	0.00	0.00	0.00	0.00	0.00
Stearic Acid	C18	0.28 ± 0.00	0.34 ± 0.01	0.30 ± 0.01	0.53 ± 0.02	0.28 ± 0.04	1.06 ± 0.04	0.35 ± 0.01	0.31 ± 0.01
Oleic Acid	C18:1t	0.00	0.01 ± 0.00	0.02 ± 0.01	0.03 ± 0.01	0.02 ± 0.01	0.04 ± 0.01	0.01 ± 0.01	0.01 ± 0.00
Oleic Acid	C18:1c	1.76 ± 0.03	2.30 ± 0.04	1.93 ± 0.02	3.42 ± 0.07	1.76 ± 0.02	7.07 ± 0.08	2.36 ± 0.04	2.07 ± 0.01
Linoleic Acid	C18:2c	3.93 ± 0.02	5.69 ± 0.04	4.70 ± 0.01	8.01 ± 0.09	4.44 ± 0.12	16.96 ± 0.28	5.84 ± 0.04	5.16 ± 0.02
Arachidic Acid	C20	0.02 ± 0.00	0.15 ± 0.01	0.04 ± 0.02	0.08 ± 0.01	0.03 ± 0.04 ± 0.00	0.12 ± 0.21	0.11 ± 0.01	0.12 ± 0.00
g-Linolenic Acid	C18:3W6	0.00	0.01 ± 0.00	0.00	0.00	0.00	0.01 ± 0.01	0.00	0.00

Gondoleic (Eicosenoic Acid)	C20:1W9	0.01 ± 0.00	0.83 ± 0.02	0.01 ± 0.00	0.02 ± 0.01	0.01 ± 0.00	0.04 ± 0.00	0.01 ± 0.00	0.01 ± 0.00
a-Linolenic Acid	C18:3W3	0.53 ± 0.01	0.00	0.65 ± 0.01	1.12 ± 0.02	0.61 ± 0.00	2.29 ± 0.03	0.84 ± 0.02	0.74 ± 0.03
Eicosadienoic Acid	C20:2W6	0.01 ± 0.00	0.01 ± 0.00	0.01 ± 0.00	0.01 ± 0.00	0.01 ± 0.00	0.02 ± 0.00	0.01 ± 0.00	0.01 ± 0.00
Behenic Acid	C22	0.02 ± 0.01	0.03 ± 0.00	0.03 ± 0.00	0.05 ± 0.00	0.03 ± 0.00	0.09 ± 0.00	0.03 ± 0.00	0.03 ± 0.00
g-Eicosatrienoic Acid	C20:3W6	0.00	0.01 ± 0.00	0.00	0.00	0.00	0.00	0.00	0.01 ± 0.00
Eicosatrienoic Acid	C20:3W3	0.00	0.00	0.00	0.00	0.00	0.00	0.00	0.00
Tricosanoic Acid	C23	0.00	0.00	0.00	0.01 ± 0.00	0.00	0.01 ± 0.00	0.00	0.00
Lignoceric Acid	C24	0.00	0.01 ± 0.00	0.01 ± 0.00	0.01 ± 0.00	0.01 ± 0.00	0.03 ± 0.00	0.01 ± 0.00	0.01 ± 0.00

Table S3: Volatile compounds identified in fermented soybean samples by GC–MS analysis. Volatile compounds identified by GC–MS analysis after 72 h of fermentation. All compounds were semi-quantified relative to the internal standard. Identification was accepted when both mass spectral matching (≥ 80%) and retention index agreement (± 20 units) were achieved. ND = not detected. PN = Penicillium nalgiovense; KM = Kluyveromyces marxianus; DH = Debaryomyces hansenii; LP = Lactiplantibacillus plantarum. ND = not detected.

**Compounds**	**Aroma**	**Control**	**PN**	**KM**	**DH**	**LP**	**PNKM**	**PNDH**	**PNLP**
**Aldehydes**									
Hexanal	Green, fresh	7.63 ± 1.42	0.33 ± 0.01	ND	ND	0.66 ± 0.10	ND	ND	ND
Benzaldehyde	Sharp, sweet, bitter, almond, cherry	6.32 ± 1.03	ND	ND	ND	3.78 ± 0.17	ND	ND	ND
Nonanal	Aldehylic, fatty, citrus	ND	0.65 ± 0.03	ND	ND	ND	ND	ND	ND
2-octenal, (E)-	Waxy, aldehylic, rose, fatty	ND	0.89 ± 0.46	ND	ND	ND	ND	ND	ND
Benzeneacetaldehyde	Green, sweet, floral	ND	1.80 ± 0.84	ND	ND	ND	ND	ND	ND

Pentanal	Fermented	ND	0.96 ± 0.05	ND	ND	ND	ND	ND	ND
**Acids**									
Hexanoic acid	Fatty, cheesy	ND	ND	ND	ND	2.42 ± 0.19	ND	ND	ND
2-methyl-propanoic acid	Acidic, sour, cheesy, dairy, buttery	ND	ND	ND	ND	ND	2.37 ± 0.90	ND	ND
3-methyl-butanoic acid	Sour, cheesy, dairy, acidic	ND	ND	ND	ND	ND	8.44 ± 1.82	ND	2.11 ± 0.35
β-phenylethyl butyrate	Sweet, floral, yeasty, strawberry	ND	ND	ND	ND	ND	4.86 ± 0.29	ND	ND
Octanoic acid	Fatty, vegetable, cheesy	ND	ND	ND	ND	1.06 ± 0.94	ND	ND	ND
**Alcohols**									

1-hexanol	Herbal	6.11 ± 0.29	ND	ND	ND	5.37 ± 0.45	ND	ND	ND
3-octanol	Earthy, mushroom, herbal	0.38 ± 0.14	ND	1.26 ± 0.06	ND	ND	1.52 ± 0.51	ND	2.88 ± 1.20
1-octen-3-ol	Mushroom, earthy, green, fungal, raw chicken	12.86 ± 1.11	25.90 ± 3.82	12.64 ± 0.60	19.02 ± 2.57	18.50 ± 3.84	ND	18.09 ± 1.54	10.19 ± 0.63
2-phenylethyl alcohol	Floral, rose	ND	2.66 ± 0.64	56.32 ± 13.32	0.51 ± 0.00	ND	103.54 ± 23.07	26.59 ± 5.50	12.59 ± 4.40
Benzyl alcohol	Floral	ND	ND	ND	ND	0.51 ± 0.07	ND	ND	ND
2-methyl-1-propanol	Alcoholic, fermented	ND	ND	ND	ND	ND	ND	3.79 ± 0.18	1.38 ± 0.32
2-methyl-1-butanol	Ethereal, fatty, greasy, cocoa	ND	ND	4.98 ± 1.97	ND	ND	ND	ND	ND
3-methyl-1-butanol	Fermented, fruity, banana	ND	ND	ND	ND	ND	5.65 ± 4.36	17.67 ± 3.29	5.79 ± 2.25
**Ketones**									

3-octanone	Fresh, herbal, mushroom	0.83 ± 0.27	7.48 ± 6.47	ND	1.71 ± 1.48	ND	ND	3.64 ± 0.38	5.67 ± 0.69
2-pentanone	Sweet, banana, fruity	ND	ND	ND	1.39 ± 0.12	ND	ND	ND	ND
2-heptanone	Cheesy	ND	0.49 ± 0.22	ND	3.38 ± 0.51	ND	ND	ND	ND
2-nonanone	Fruity	ND	ND	ND	3.01 ± 0.50	ND	ND	1.91 ± 0.51	ND
2,3-butanedione	Buttery, sweet, creamy	ND	ND	ND	ND	0.70 ± 0.15	ND	ND	ND
Acetoin	Sweet, buterry, creamy, dairy, milky, fatty	ND	0.39 ± 0.03	ND	ND	8.68 ± 0.39	ND	ND	0.95 ± 0.17
**Esters**									
Ethyl acetate	Ethereal, fruity, sweet	ND	ND	ND	0.31 ± 0.02	ND	ND	ND	ND

Linoleic acid ethyl ester	Fatty	ND	ND	ND	1.68 ± 9.07	ND	3.62 ± 1.44	ND	10.12 ± 3.77
Hexadecanoic acid, ethyl ester	Waxy, fruity, creamy, milky, buttery	ND	ND	ND	ND	ND	2.29 ± 0.30	ND	7.65 ± 1.28
Ethyl oleate	Fatty, tallow, oily, buttery, waxy, milky	ND	ND	ND	ND	ND	3.15 ± 2.27	ND	8.60 ± 2.75
2-phenylethyl ester acetic acid	Floral, rose sweet, honey, fruity	ND	ND	99.78 ± 12.78	ND	ND	7.93 ± 4.51	ND	ND
**Furans**									
2-pentyl-furan	Green, earthy, beany	4.42 ± 0.93	1.16 ± 0.37	2.04 ± 0.43	1.80 ± 0.18	1.67 ± 0.23	ND	1.48 ± 0.07	0.63 ± 0.17
**Volatile phenols**									
2-methoxy phenol	Smoky, spicy	ND	0.23 ± 0.20	ND	ND	ND	ND	0.65 ± 0.12	ND
4-ethyl-phenol	Smoky, savoury, bacon, ham	ND	ND	ND	ND	ND	15.69 ± 7.70	ND	22.62 ± 4.05

Table S4 Odour activity values (OAVs) of identified volatile compounds in fermented soybean samples after 72 h. OAV = odour activity value (calculated as compound concentration divided by odour threshold). PN = Penicillium nalgiovense; KM = Kluyveromyces marxianus; DH = Debaryomyces hansenii; LP = Lactiplantibacillus plantarum. Compounds with OAV > 1 are considered potentially aroma-active.

Compound	Sample	Concentration (µg/g)	Odour Threshold (µg/g)	OAV
Hexanal	Control	7.63	4.5	1.70
Hexanal	PN	0.33	4.5	0.07
Hexanal	LP	0.66	4.5	0.15
Benzaldehyde	Control	6.32	350	0.02
Benzaldehyde	LP	3.78	350	0.01
Nonanal	PN	0.65	0.9	0.72
2-octenal, (E)-	PN	0.89	0.45	1.98
Benzeneacetaldehyde	PN	1.8	2.7	0.67
Pentanal	PN	0.96	4.5	0.21
Hexanoic acid	LP	2.42	378	0.01
2-methyl-propanoic acid	PNKM	2.37	3.6	0.66

3-methyl-butanoic acid	PNKM	8.44	3.6	2.34
3-methyl-butanoic acid	PNLP	2.11	3.6	0.59
β-phenylethyl butyrate	PNKM	4.86	1.8	2.70
1-hexanol	Control	6.11	630	0.01
1-hexanol	LP	5.37	630	0.01
3-octanol	PNKM	1.52	270	0.01
3-octanol	PNLP	2.88	270	0.01
1-octen-3-ol	Control	12.86	0.9	14.29
1-octen-3-ol	PN	25.9	0.9	28.78
1-octen-3-ol	KM	12.64	0.9	14.04
1-octen-3-ol	DH	19.02	0.9	21.13
1-octen-3-ol	LP	18.5	0.9	20.56
1-octen-3-ol	PNDH	18.09	0.9	20.10
1-octen-3-ol	PNLP	10.19	0.9	11.32
2-phenylethyl alcohol	PN	2.66	10	0.27
2-phenylethyl alcohol	KM	56.32	10	5.63

2-phenylethyl alcohol	DH	0.51	10	0.05
2-phenylethyl alcohol	PNKM	103.54	10	10.35
2-phenylethyl alcohol	PNDH	26.59	10	2.66
2-phenylethyl alcohol	PNLP	12.59	10	1.26
3-octanone	Control	0.83	0.12	6.92
3-octanone	PN	7.48	0.12	62.33
3-octanone	DH	1.71	0.12	14.25
3-octanone	PNDH	3.64	0.12	30.33
3-octanone	PNLP	5.67	0.12	47.25
2-heptanone	DH	3.38	180	0.02
2-nonanone	DH	3.01	180	0.02
2-nonanone	PNDH	1.91	180	0.01
2,3-butanedione	LP	0.7	0.1	7.00
Acetoin	PN	0.39	0.8	0.49
Acetoin	LP	8.68	0.8	10.85
Acetoin	PNLP	0.95	0.8	1.19

Ethyl acetate	DH	0.31	5	0.06
Linoleic acid ethyl ester	DH	1.68	1	1.68
Linoleic acid ethyl ester	PNKM	3.62	1	3.62
Linoleic acid ethyl ester	PNLP	10.12	1	10.12
Hexadecanoic acid, ethyl ester	PNKM	2.29	0.4	5.73
Hexadecanoic acid, ethyl ester	PNLP	7.65	0.4	19.13
Ethyl oleate	PNKM	3.15	0.25	12.60
Ethyl oleate	PNLP	8.6	0.25	34.40
2-phenylethyl ester acetic acid	KM	99.78	225	0.44
2-phenylethyl ester acetic acid	PNKM	7.93	225	0.04
2-pentyl-furan	Control	4.42	5.4	0.82
2-pentyl-furan	PN	1.16	5.4	0.21
2-pentyl-furan	KM	2.04	5.4	0.38
2-pentyl-furan	DH	1.8	5.4	0.33
2-pentyl-furan	LP	1.67	5.4	0.31
2-pentyl-furan	PNDH	1.48	5.4	0.27

2-pentyl-furan	PNLP	0.63	5.4	0.12
2-methoxy phenol	PN	0.23	9	0.03
2-methoxy phenol	PNDH	0.65	9	0.07
4-ethyl-phenol	PNKM	15.69	162	0.10
4-ethyl-phenol	PNLP	22.62	162	0.14

Table S5 Targeted LC–MS screening of selected Penicillium-associated secondary metabolites in Penicillium nalgiovense (PN) monoculture fermentation after 72 h. Compounds screened included hydroxy-aspergillic acid, neohydroxy-aspergillic acid, neoaspergillic acid, aspergillic acid, and patulin (putative). ND = not detected. Results are shown for biological replicates.

**Compound**			**Detection**
	**R1**	**R2**	
Hydroxy-aspergillic acid	ND	ND	Not detected
Neohydroxy-aspergillic acid	ND	ND	Not detected
Neoaspergillic acid	ND	ND	Not detected
Aspergillic acid	ND	ND	Not detected
Patulin (putative)	ND	ND	Not detected
